# A Multivariate Dynamic Spatial Factor Model for Speciated Pollutants and Adverse Birth Outcomes

**DOI:** 10.3390/ijerph14091046

**Published:** 2017-09-11

**Authors:** Kimberly A. Kaufeld, Montse Fuentes, Brian J. Reich, Amy H. Herring, Gary M. Shaw, Maria A. Terres

**Affiliations:** 1 Los Alamos National Laboratory, P.O. Box 1663, Los Alamos, NM 87545, USA; 2 Department of Biostatistics and Statistics and Operations Research, Virginia Commonwealth University, Richmond, VA 23284, USA; mfuentes@vcu.edu; 3 Department of Statistics, North Carolina State University, Raleigh, NC 27695, USA; bjreich@ncsu.edu; 4 Department of Biostatistics, University of North Carolina, Chapel Hill, NC 27599, USA; herring@unc.edu; 5 Division of Neonatology, Department of Pediatrics, Stanford University School of Medicine, Stanford, CA 94305, USA; gmshaw@stanford.edu; 6 The Climate Corporation, San Francisco, CA 94103, USA; mterres.stat@gmail.com

**Keywords:** multivariate, spatiotemporal, birth defects, pollutants, factor analysis

## Abstract

Evidence suggests that exposure to elevated concentrations of air pollution during pregnancy is associated with increased risks of birth defects and other adverse birth outcomes. While current regulations put limits on total PM${}_{2.5}$ concentrations, there are many speciated pollutants within this size class that likely have distinct effects on perinatal health. However, due to correlations between these speciated pollutants, it can be difficult to decipher their effects in a model for birth outcomes. To combat this difficulty, we develop a multivariate spatio-temporal Bayesian model for speciated particulate matter using dynamic spatial factors. These spatial factors can then be interpolated to the pregnant women’s homes to be used to model birth defects. The birth defect model allows the impact of pollutants to vary across different weeks of the pregnancy in order to identify susceptible periods. The proposed methodology is illustrated using pollutant monitoring data from the Environmental Protection Agency and birth records from the National Birth Defect Prevention Study.

## 1. Introduction

The association of air pollution exposure and adverse pregnancy outcomes has been found in a number of recent studies. Zeiger and Beaty [1] mention that both genetic and environmental factors play important roles in oral clefts. In a fetal study on rats, exposure to ozone and carbon monoxide produced a skeletal malformation in animals [2,3]. Air pollution was also found to contribute to the development of skeletal malformations via toxicity and biologic mechanisms [4].

Several studies have found adverse effects of air pollution exposure during pregnancy and birth defects [4,5]. In a case control study of women in Los Angeles, Ritz et al. [4] found an association of air pollution, CO and ozone, O${}_{3}$, exposure and birth defects including oral clefts. In another study, the amount of exposure to O${}_{3}$ during the first and second month of pregnancy was found to increase the risk of oral clefts [6], one of the types of orofacial clefts. In one study conducted in China, environmental factors were found to be more strongly associated with cleft lip and/or palate [7]. In another study in Texas, there was a weak association between particulate matter, PM${}_{10}$, and isolated cleft lip with or without cleft palate [5]. A study conducted in the California National Birth Defects Prevention Study (NBDPS) region, Padula et al. [8] found associations between traffic density and cleft lip with or without palate, but no associations with particulate matter and birth defects.

A common statistical challenge in these epidemiological studies is estimating a mother’s exposure with sparse monitor locations [9]. Studies have recommended including only mothers within 6 km or 12 km of a monitoring station [10]. However, in many birth defect studies, the mother is often assigned to the nearest air pollution monitor [4,5,11]. As there are approximately 40 stations in all of California, the distance to a monitor and quality of the proxy can vary dramatically depending upon where a woman resides. In our study, we interpolate the factors of the pollutants using a geospatial approach, Gaussian processes, at the mother’s location rather than only to the closest location. This allows us to get a more accurate idea of the the impact of the factors at different times of the mothers’ pregnancy and can help account for the maternal proximity to high areas of pollution.

In our study, we investigate the effects of speciated components, the particles that compose particulate matter, PM${}_{2.5}$, to help determine which components have a more significant impact on cleft birth defects. Earlier studies only used O${}_{3}$ and total PM${}_{2.5}$ [4,11]. We extend the results to account for several pollutants during critical gestation periods when women are more susceptible to pollutants. Exposure is usually aggregated to the month or trimester, and only a few studies have looked at exposure at the weekly level in association with selected birth defects [12,13,14]. In the weekly model, a Bayesian hierarchical framework was developed to assess critical windows of exposure between weeks 3–8. It used weekly averages during embryonic development and identified windows using a non-Gaussian and spatial-temporal associations. The model, however, did not look at multiple pollutants simultaneously.

Our model expands upon spatial dynamic factor analysis recently proposed in the literature by including a multivariate dimension of factors while simultaneously modeling the health effects. The spatial dynamic factor analysis developed by Lopes et al. [15] differs in two key ways. First, their approach is presented for univariate spatio-temporal data, whereas ours allows for consideration of multiple spatio-temporal processes (pollutants) jointly. Secondly, the factors in their model are purely temporal and thus only allow for examination of spatial variation in the factor loadings, not in the factors themselves. In contrast, the Bayesian spatial confirmatory factor analysis for climate is illustrated by Neeley et al. [16] is both multivariate and produces a spatial factor. In particular, the spatial factor represents a weighted average of the original multivariate spatial processes with spatially smooth factor loadings. However, this analysis was developed for areal data using a conditionally autoregressive (CAR) structure and does not capture temporal evolution in the processes. Our proposed model merges ideas from each of these papers in order to produce a multivariate spatial dynamic factor model with spatio-temporal factors that evolve coherently in time, while simultaneously relates the factor to the health outcomes.

In our model, the spatio-temporal factors are utilized as predictors in a model for human health (e.g., risk of birth defect). The work of Jandarov and Szpiro [17] has a similar goal, but utilizes principal components (PC) in lieu of a factor model. The PC scores vary spatially and are regressed on geographical covariates with a Gaussian process error allowing for interpolation at new locations. The framework does not, however, incorporate any temporal structure into the model. In contrast, our approach is fit in a Bayesian framework and produces interpretable spatial factor loadings and temporal dynamics. In this paper, we introduce a spatial dynamic factor model using Bayesian multivariate (binary) probit regression. The model accounts for multiple pollutants in the factor component model at the critical times in a mother’s pregnancy, weeks 3–8.

The analysis is carried out using one center’s data from the National Birth Defects Prevention Study (NBDPS), a case-control study in California’s San Joaquin Valley. It consists of cleft defects from 2002 to 2008 along with parental demographic and birth outcome information for all births. Weekly averages of the speciated PM${}_{2.5}$ based upon the mother’s residential location and dates of pregnancy, are estimated from the Air Quality System (AQS) monitoring data. Exposure estimates are obtained from the Environmental Protection Agency’s monitoring stations, Speciation Trends Network (STN) and Interagency Monitoring for Protected Visual Environments (IMPROVE) that provide ambient monitoring station data.

In Section 2, we describe the data used in the analysis. Section 3 introduces the statistical model. In Section 4, the spatial dynamic factor model is applied to the California birth defects dataset including a short sensitivity analysis of the factors. In Section 5, we close with conclusions and discussions.

## 2. Data Description

### 2.1. National Birth Defects Data

The oral cleft defects data in California is from the National Birth Defects Prevention Study, a population-based, multi-center case control study of birth defects [18]. It includes information on live births from 2002 to 2008 in California. The methods are described in Reefhuis et al. [18]. Cases included live births, and controls are live births without any known birth defects, identified randomly from selected hospitals in California. We analyzed both cleft lip and cleft palate defects. There were a total of 208 cleft lip or cleft palate defects reported and 358 controls. Cases listed as cleft palate include a cleft lip and cleft palate defect, similarly cleft lip includes a cleft lip and cleft palate defect.

As part of the study, women reported their complete residential history during pregnancy. Based upon this information, all residences were geocoded at the first eight weeks of pregnancy to assign exposure levels. The NBDPS protocol estimates each woman’s date of conception based upon the estimated date of delivery—in other words, the due date that the woman received from her physician, which was reported at the study interview. The estimated date of conception is used to identify exposure levels during gestation periods, weeks 3–8, the pertinent embryologic period of lip and palate formation. In the case that a woman had more than one residence, the mother was assigned a different exposure level based upon the residing location. We also used potential confounder variables displayed in Table 1: sex of the infant, maternal education (high school, some college and college), maternal age classifications (19 and under, 20–24, 25–29, 30–34, and 35 and older), high blood pressure during pregnancy, smoking while pregnant and alcohol use while pregnant.

### 2.2. Pollution Data

The AQS monitoring data collected by the Environmental Protection Agency (EPA) are available for California from 2003 to 2007. Speciated PM${}_{2.5}$ values in micrograms per cubic meter (ug/m${}^{3}$) are collected to construct a map of the pollutant sites in the state of California. Although the birth defects study does not cover all of California, we use all the station data to help account for a mother’s mobility during the study period. There were approximately 15% of the women moved during the study to areas outside of the initial study area. In some cases, areas moved to are not in the original study area, thus it is important to include stations from outside the study region in this case including all stations in California. These data consist of measurements collected every three to six days by the EPA’s Interagency Monitoring for Protected Visual Environments (IMPROVE) and STN sites. The IMPROVE monitors are located in more remote areas, while the STN monitors are located in more urban areas. The two monitoring networks have 40 monitors, and 20 are in the study region, combining the two data sources provides good coverage of California. The speciated components selected for the study are Ammonium, Nitrate, Sulfate, Total Carbon, Calcium, Iron, Potassium, Silicon, and Sulfur.

**Figure 1:**
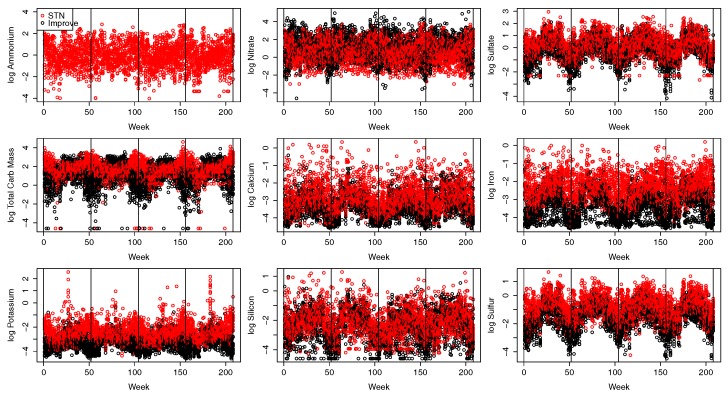
STN and IMPROVE stations speciated components for the years 2003–2006 weekly averages for each site where the vertical lines separate each year. Ammonium is only collected at STN sites.

The speciated components are combined by a dynamic spatial factor analysis, and resulting factors are interpolated using bivariate spline interpolation, a geospatial technique, to each mother in the study. The interpolated factors represent the ambient concentration at the mother’s residence. The weekly factor averages for gestational weeks three through eight are considered based on embryonic timing of an association with orofacial clefts. The nine speciated components, at each site, are averaged weekly from 2003 to 2006. All sites pollutant values are shown on the log scale in Figure 1. A clear seasonal pattern can be found across the speciated pollutants, which is captured in the dynamic model.

The speciated pollutants can be highly correlated. In particular, from Table 2, we see that Ammonium and Nitrate are highly correlated with a value of 0.96 as well as Sulfur and Sulfate correlation value of 0.97. Due to the high correlation among the pollutants, the speciated components need to be modeled together rather than separately.

## 3. Multivariate Spatio-Temporal Factor Model for Pollution

In this section, we describe our model to relate the speciated particulate matter mixture with birth defect outcomes. Our model expands upon spatial dynamic factor analysis recently proposed in the literature by including a multivariate dimension of factors while simultaneously modeling the health effects. In Section 3.1, we give the spatiotemporal factor model for speciated PM${}_{2.5}$. In Section 3.2, we give the model to relate the latent particulate matter factors with the health outcomes. Although the aspects of the model are described separately, they are fit simultaneously using a Bayesian hierarchical model, the pollutant model is evaluated at the first stage and the birth defect model is estimated at the second stage in one algorithm. The computational algorithm with the priors for the model is described in the Appendix.

### 3.1. Factor Model for Speciated PM${}_{2.5}$

The factor model for speciated PM${}_{2.5}$ expands upon the spatial dynamic factor analysis presented for univariate spatio-temporal data, recently proposed in the literature by Lopes et al. [15]. The extension of the proposed model allows for consideration of multiple spatio-temporal processes (pollutants) jointly as well as providing spatial variation in the factors. We use the notation similar to Lopes et al. [15] as follows.

Let $Y_{tp}\left(s\right)$ be the observation at location *s* on day t=1,⋯,T, for pollutant p=1,⋯,P, and $Y_{t}\left(s\right)={\left[Y_{t1}\left(s\right),\cdots ,Y_{tP}\left(s\right)\right]}^{T}$. The high correlations between pollutants, as shown in Section 2.2, and spatial variability in the pollutant sources favors dimension reduction in the number of pollutants. We use M≤P latent factors to represent variation in the *P* pollutants. Let $\delta_{t}\left(s\right)={\left[\delta_{t1}\left(s\right),\cdots ,\delta_{tM}\left(s\right)\right]}^{T}$ be the latent vector of factors for time *t* at location *s*.

Our model is a dynamic linear model with observation and evolution equations:

(1) $$\begin{array}{cc} Y_{t}\left(s\right) & =\mu_{t}\left(s\right)+\Lambda \left(s\right)\delta_{t}\left(s\right)+\epsilon_{t}\left(s\right),\\ \delta_{t}\left(s\right) & =\Gamma \left(s\right)\delta_{t-1}\left(s\right)+w_{t}\left(s\right). \end{array}$$

In Equation (1), $\mu_{t}\left(s\right)={\left[\mu_{t1}\left(s\right),\cdots ,\mu_{tP}\left(s\right)\right]}^{T}$ is the *P*-vector of means that vary slowly over time to capture seasonality (e.g., Figure 1); Λ(s) is the P×M factor loading matrix that relates the latent factors to the observed pollutant concentrations; Γ(s) is the M×M matrix that controls the propagation of the latent factors; $\epsilon_{t}\left(s\right)={\left[\epsilon_{t1}\left(s\right),\cdots ,\epsilon_{tP}\left(s\right)\right]}^{T}$ are errors with $\epsilon_{tj}\left(s\right)∼N\left(0,\sigma^{2}\right)$; and $w_{t}\left(s\right)={\left[w_{t1}\left(s\right),\cdots ,w_{tM}\left(s\right)\right]}^{T}$ captures the spatial process at location *s* using a zero mean Gaussian process with variance $V\left[w_{tl}\left(s\right)\right]=s_{w}^{2}$ and spatial correlation cor$\left[w_{tl}\left(s\right),w_{tl}\left(s^{\prime }\right)\right]=R(||s-s^{\prime }{\left|\right|}_{j},\phi_{w})$ denoted $w_{tl}∼GP\left[0,s_{w}^{2}R\left(\phi_{w}\right)\right]$.

The effect of factor *f* on pollutant *p* is determined by the (p,f) element of Λ(s), denoted by $\lambda_{pf}\left(s\right)$. To ensure identification, we fix $\lambda_{pf}\left(s\right)=0$ for f<p and $\lambda_{pf}\left(s\right)>0$ for p=1,⋯,M. To induce spatial smoothness in the loadings, we model the elements $\log[\lambda_{pp}\left(s\right)]$ and $\lambda_{pf}\left(s\right)$ for f>p as realizations from a Gaussian process, $GP[0,s_{\lambda_{pf}}^{2}R\left(\phi_{\lambda_{pf}}\right)]$.

The propagation matrix Γ(s) is taken to be diagonal with diagonal elements $\gamma_{1}\left(s\right),\cdots ,\gamma_{M}\left(s\right)$ for identification in the case where factors are rotated. Spatial smoothness in the evolution processes $\gamma_{p}\left(s\right)$ is again desirable, so we model them as a Gaussian process, $\gamma_{f}∼GP\left[0,s_{\Gamma_{f}}^{2}R\left(\phi_{\Gamma_{f}}\right)\right]$. We constrain the factor evolution coefficients, $\gamma_{f}$, to the interval (−1,1) to ensure stationarity of the temporal processes using a truncated Gaussian process [19]. Finally, to ensure spatial smoothness in the dynamic factors, the initial values $\delta_{0}$ are each assumed to be a realization from a Gaussian process, $\delta_{f,0}∼GP\left[0,s_{\delta_{f}}^{2}R\left(\phi_{\delta_{f}}\right)\right]$. We have assumed that the μ vector will center the pollutant quantities such that the means of the factors, factor loadings, and factor evolution coefficients will be 0.

In summary, an observation $Y_{tp}\left(s\right)$ of pollutant *p* at location *s* and time *t* will be centered at $\mu_{tp}\left(s\right)$ plus some linear function of the *M* factors at location *s* and time *t*. These factors will be spatially smooth and evolve in time therefore accounting for the underlying correlation structure. The mutual dependence on the same *M* factors builds dependence between the *P* pollutants. Imagine we have M=2 sources of pollution, say traffic and factories. We have no direct measures of these sources, $\delta_{t1}\left(s\right)$ and $\delta_{t2}\left(s\right)$, so we instead relate these latent factors to speciated pollution levels, say organic carbon, elemental carbon, nitrate and sulfate, which *can* be measured. At any location, the measured pollutant level will depend on the pollutant sources as determined by Λ(s). Through the use of spatially varying factor loadings, the current model allows, for example, the amount of nitrate in traffic pollution to vary spatially. In one area, the factor loading may be high, indicating that the traffic pollution is particularly high in nitrate, while, in another area, it may be low indicating that traffic pollution is relatively low in nitrate. In the case of spatially constant factor loadings, the model would suggest that traffic produces a fixed amount of nitrate relative to its abundance regardless of location.

### 3.2. Tailoring the Pollutant Model to CA Pollutant Data

The general spatial dynamic factor model described in Section 3.1 is augmented to fit the ambient air pollutant data in California described in Section 2. The pollutant data are from two observation networks: STN for urban sites and IMPROVE (IMP) for rural sites. We allow the mean and error variances to differ based upon the pollutants sites in the model by accounting for the two networks in Equation (1) as follows:

$$\mu_{tp}\left(s\right)=\left\{\begin{array}{c} {\bar{\mu }}_{0p}\;\mathrm{if}\;s\;\mathrm{is}\;\mathrm{an}\;\mathrm{IMPROVE}\;\mathrm{site},\\ {\bar{\mu }}_{0p}+{\bar{\mu }}_{1p}\;\mathrm{if}\;s\;\mathrm{is}\;a\;\mathrm{STN}\;\mathrm{site}, \end{array}\right.$$

and $\epsilon_{t,p}\left(s\right)∼N\left[0,\sigma_{p}^{2}\left(s\right)\right],$ where

$$\sigma_{tp}^{2}=\left\{\begin{array}{c} \sigma_{STN,p}^{2}\\ \sigma_{IMP,p}^{2}. \end{array}\right.$$

The speciated components, accounting for the two observation networks, are combined via the dynamic spatial factor analysis described in Section 3.1. The model reduces the speciated components to only a few factors. The resulting factors from the model are then interpolated to the mother’s location based upon the prior distributions (see Appendix A) of the factors. The weekly factors associated with each woman during gestational weeks 3 through 8, corresponding to the embryonic timing for cleft defects, are consequently used in the birth defect model as described below.

### 3.3. Birth Defect Model

The California birth defect data has two binary responses denoted

$${\tilde{Y}}_{i1}=\left\{\begin{array}{cc} 0, & \mathrm{no}\;\mathrm{cleft}\;\mathrm{palate}\;\mathrm{defect},\\ 1, & \mathrm{cleft}\;\mathrm{palate}\;\mathrm{defect}\;\mathrm{for}\;\mathrm{individual}\;i, \end{array}\right.$$

$${\tilde{Y}}_{i2}=\left\{\begin{array}{cc} 0, & \mathrm{no}\;\mathrm{cleft}\;\mathrm{lip}\;\mathrm{defect},\\ 1, & \mathrm{cleft}\;\mathrm{lip}\;\mathrm{defect}\;\mathrm{for}\;\mathrm{individual}\;i. \end{array}\right.$$

The multiple binary responses are modeled using probit regression to induce conjugacy to simultaneously fit the factor and birth defect models in the Bayesian hierarchical setting. The probability of having a cleft defect is modeled assuming a set of latent variables, $Z_{i}=\left(Z_{i1},Z_{i2}\right)$ such that ${\tilde{Y}}_{ij}=I\left(Z_{ij}>0\right)$ and

$$\begin{array}{c} Z_{i}=\beta^{T}x_{i}+\sum_{m=1}^{M}\sum_{\ell =3}^{L=8}w_{\ell m}^{T}\delta_{i\ell m}+\epsilon_{i}. \end{array}$$

The vector $x_{i}$ contains individual-level covariates such as maternal age (Table 1), β is a matrix of regression coefficients specific to the birth defect which relates the covariates, i.e., fetus’ gender, mother’s age, race, etc., to the latent response, $w_{\ell m}^{T}$ represents the effect of exposure to factor *m* during gestation week *ℓ*, $\delta_{i\ell m}$ is the value of factor *m* for mother *i* at gestation week *ℓ*, i.e., a specific value of the latent factors, $\delta_{tj}\left(s\right)$, from Equation (1) and $\epsilon_{i}$~N(0,Ω). The latent variables, $Z_{i}$, are used to define the outcomes when ${\tilde{Y}}_{ij}=1$, i.e., defect *j* was observed if $Z_{ij}>0$, whereas if a control was observed, $Z_{ij}<0$. This lends itself to interpretation of the probability of a defect, P$\left(Z_{ij}>0\right)$, or if a control is observed P($Z_{ij}<0$). Therefore, the probability of ${\tilde{Y}}_{ij}=1$, P(${\tilde{Y}}_{ij}=1$) is equal to $\Phi (\beta^{T}x_{i}+w\delta_{i})$, where Φ is the standard normal distribution function that increases with the linear predictor $\beta^{T}x_{i}+w\delta_{i}$. For example, if one element of β is positive, then an increase in the corresponding covariate increases the probability of a defect. On the other hand, if one element of β is negative, then a decrease in the corresponding covariate decreases the probability of a defect.

To account for the effects of pollutants at different gestation times of the pregnancy, the temporal covariates from the M factors are accounted for using a Gaussian process

$$w_{m}^{T}∼\mathrm{GP}\left[0,R\left(\phi \right)\right],$$

where the coefficients are allowed to change smoothly across pregnancy weeks. In this case, $w_{m}^{T}$ are the coefficients for each of the $Z_{i}$ clefts (cleft lip and cleft palate) weekly measurements of pollutant index M (factors). For example, if one element of w is positive, then an increase in the corresponding pollutant factor increases the probability of a defect and vice versa.

As this is a fully Bayesian approach, the model is specified by assigning prior distributions to the model parameters. The mean coefficients are β∼N(0,I). In this model, cleft lip and cleft palate can occur at the same time, and are not mutually exclusive. The correlation coefficient between defects is ϕ=0.51. To account for the correlation structure, we use an inverse Wishart for the covariance structure, Ω∼IW(Σ,ν), where $\Sigma =I_{2}$ and ν=2.

The self reported confounders in $x_{i}$ include indicators for maternal age categories 19 and under, 20–24, 25–29, 30 and older; maternal education categorized as less than high school, high school diploma/equivalency and/or some college or trade school and college graduate or advanced degree; binary indicators of maternal smoking during pregnancy categorized as yes/no; maternal alcohol consumption during the pregnancy as any/none; maternal prediabetes, high blood pressure; and sex of the fetus.

## 4. Results

### 4.1. Impacts on Oral Cleft Risks

To assess the impacts on oral clefts, we look at the effect of the covariates and then analyze the factor pollutants based upon weekly exposure to a mixture of speciated pollutants mentioned in Section 2.2. The final dataset we analyze includes 536 observations with the results based upon 20,000 draws from the posterior distribution after a burn-in period of 5000 draws. These values were determined by trace plots for stability in the chains.

**Figure 2:**
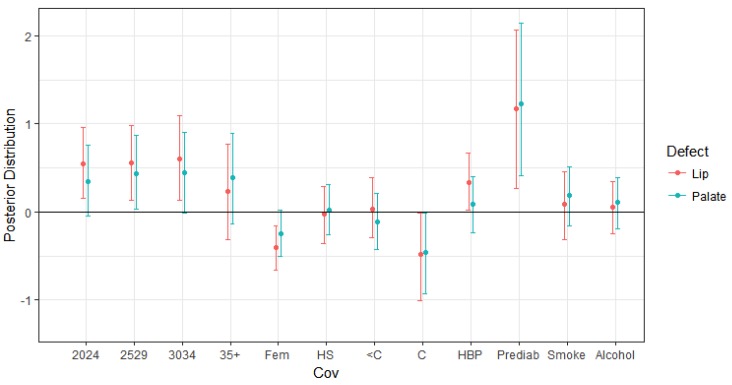
Covariate effects for the multivariate binary probit model. The posterior means (dots) and 95% credible intervals (lines) for a change in cleft defects with a one-standard deviation increase in maternal age, education, maternal smoking, and maternal alcohol consumption in the California National Birth Defects Prevention Study, 2003–2006. The labels from left to right are maternal age 20–24 vs. 19 and under (2024), 25–29 vs. 19 and under (2529), 30–34 vs. 19 and under (3034), 35+ vs. 19 and under (35+), fetus sex-female (Fem), maternal high school education vs. less than high school (HS), some college vs. less than high school (<C), college graduate vs. less than high school (C), high blood pressure (HBP), maternal prediabetes (Prediab), maternal smoking (Smoke), maternal alcohol (Alcohol).

Table 3 and Figure 2 display the posterior summaries for the effect of the covariates. For example, if one of the coefficients is positive, it increases the probability of a defect, whereas a negative coefficient decreases the probability of a defect. The age of the mother increases the the probability of cleft defects, both lip and palate. There is a general trend that any age group over 19 has a higher risk of defects compared to the baseline group of ages 19 and under, with cleft lip defects having a slightly higher probability for ages 20–34. The age group 35 and over also has an increased probability of defects; however, it is not significant for either cleft lip or cleft palate defect. Another covariate that increases the probability either a cleft lip or cleft palate defect is mothers with prediabetes. There is also an increase in the probability of a cleft palate defect if the mother has high blood pressure. However, it was not found to be a significant factor for cleft palate. The covariates that portray a decrease in the probability of a defect include the mother having a college education compared to less than a high school education for both cleft lip and cleft palate. If the sex of the fetus is female, the probability of a cleft lip defect is lower compared to a male fetus. Interestingly, smoking and alcohol use did not contribute significantly to cleft lip or cleft palate defect, which may be partially due to mothers in the study self reporting substance use.

### 4.2. Model Comparison

We compare M=1,**2**, and **3** factor models using the deviance information criterion (DIC) of Spiegelhalter et al. [20]. DIC is based upon the posterior distribution of the deviance statistic, $D\left(\theta \right)=-2\log(p\left(\tilde{Y}|\theta \right)),$ where $p(\tilde{Y}|\theta )$ represents the likelihood of the observed data given the vector of all model parameters, θ. The posterior expectation of the deviance, $\bar{D},$ describes the fit of the model to the data and $p_{D}=\bar{D}-D\left(\bar{\theta }\right)$ describes the complexity of the model based upon an effective number of parameters. DIC is defined as DIC = $\bar{D}+p_{D}$, the lower the value of DIC the better the fit.

Comparison of the models show that DIC is lowest for the one-factor model (DIC = 1624.06) compared to the two-factor (DIC = 1628.15) and three-factor (DIC = 1629.98) models. Although there was not a large difference in DIC for the three models, the impact of pollutants each week are more clearly defined in the one-factor model and therefore we proceed with the M=1 to present the results. To further see how the model fits, we checked the predicted probabilities for the one factor model to see how well the model classified the defects. The percent of defects that were correctly identified as defects, $P(\hat{Y}=1|Y=1)$, came to approximately 92%, resulting in only 8% of the defects classified incorrectly.

### 4.3. Latent Factor Results

**Figure 3:**
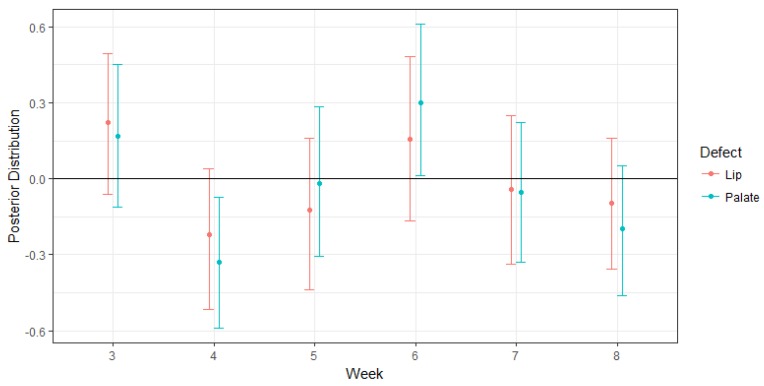
Weekly association of latent pollutants factors ($w_{\ell m}$) for the one-factor model. The effects are summarized by their posterior means (dots) and 95% credible intervals (vertical lines) for a change in cleft defects with a one-standard deviation increase in the mixture of pollutant exposure during weeks 3 through 8 post-conception in the National Birth Defects Prevention Study, 2003–2007. The factor pollutants are adjusted for maternal age, education, maternal smoking, and maternal alcohol use. Weeks 4, 5, 7 and 8 are associated with a slightly lower probability of having a cleft palate defect, whereas exposure during weeks 3 and 6 is associated with a slight increase in the probability of having a cleft palate defect.

The weekly association of the latent pollutant factors is shown in Figure 3. The mixture of pollutants from the factor model shows that weeks 4 and 6 have the largest impact on defects. Exposure during weeks 4, 5, 7 and 8 is associated with a slightly lower probability of having a cleft palate defect, whereas exposure during weeks 3 and 6 is associated with a slight increase in the probability of having a cleft palate defect, particularly higher for cleft palate in week 6. There was not a clear week where the weekly pollutants for cleft lip had a significant increase; however, it follows a similar pattern as the cleft palate.

**Figure 4:**
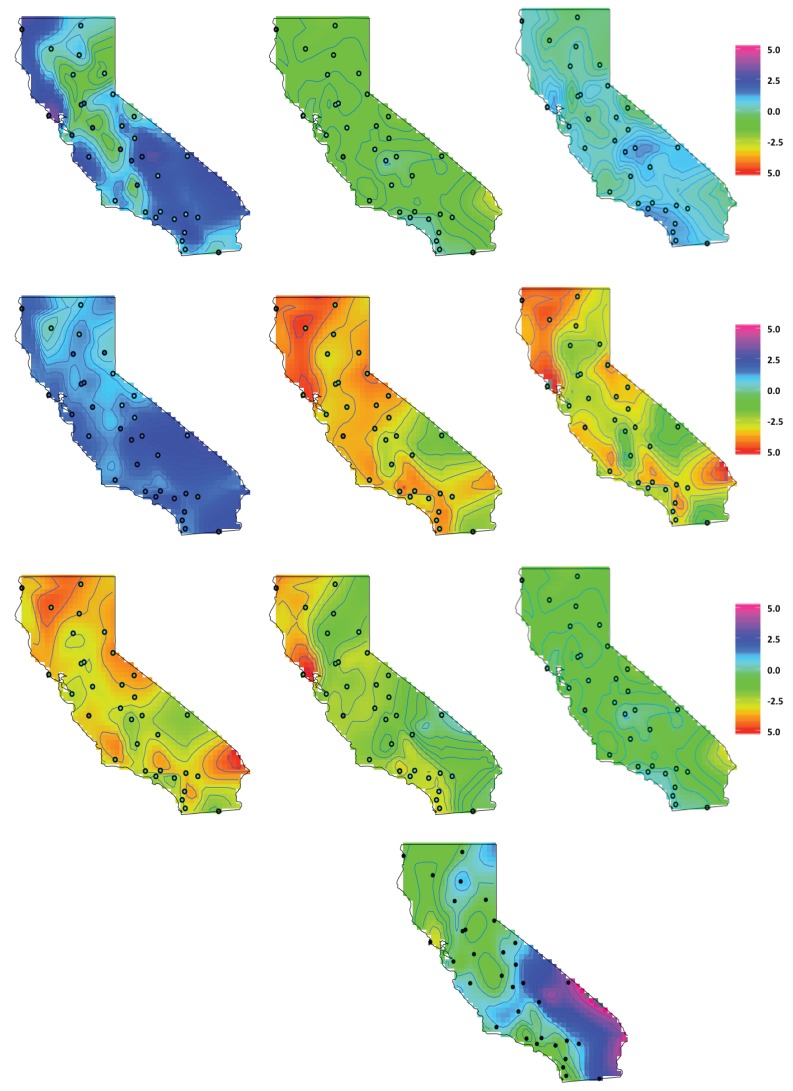
The daily mean standardized latent factors, $\left(\delta -\mu_{\delta }\right)/\sigma_{\delta }$, of δ(s) are shown in the bottom row. The standardized pollutants, Y, are (**1st row**) Ammonium, Nitrate, Sulfate, (**2nd row**) Total Carbon Mass, Calcium, Iron, (**3rd row**) Potassium, Silicon, Sulfur and (**4th row**) the Latent Factors. The dots represent the STN and IMPROVE monitoring sites with actual data values shaded in the dots.

To gain a better understanding of the impact the individual pollutants have on clefts for the one factor model, the estimated pollutants and the average of all the factor components, δ(s), are compared. We note that, although the study region was conducted in central California, we present estimates for the entire state to help account for the mother’s movement during the study. Approximately 15% of the women moved during the duration of the study to areas outside of the initial study area, with the majority of the women moving to the San Francisco Bay area, Los Angeles and the San Diego area. These are high density population areas that generally have high concentrations of pollutants. To gain a more complete understanding of the individual pollutant effects, we standardized the estimated pollutants in CA. The daily mean standardized estimated pollutants, $\left(Y_{p}-\mu_{Y_{p}}\right)/\sigma_{Y_{p}}$ is displayed in Figure 4. In the main study area, Central Valley of California, the highest concentrations of speciated pollutants are identified as Calcium, Total Carbon Mass, Sulfate and Potassium. Total carbon mass has a more equal concentration of pollutant values across the state of California. In high density areas, such as Los Angeles and San Diego, there is a general pattern of higher concentrations of Ammonium, Nitrate, Sulfate, and Sulfur. If the mother moved during the study period to any of these areas, the fetus would likely have been exposed to higher concentrations of pollutants. In the southeast corner of the state near the Nevada border, near Las Vegas on the Nevada side, higher concentrations of Total Carbon, Potassium, Iron, Sulfate and Ammonium are present, although not as high as the Bay area and Los Angeles. The factor components (δ’s) show a higher concentration of pollutants in the mid to southeastern side of California, part of the study region, and the coastal area near San Francisco.

**Figure 5:**
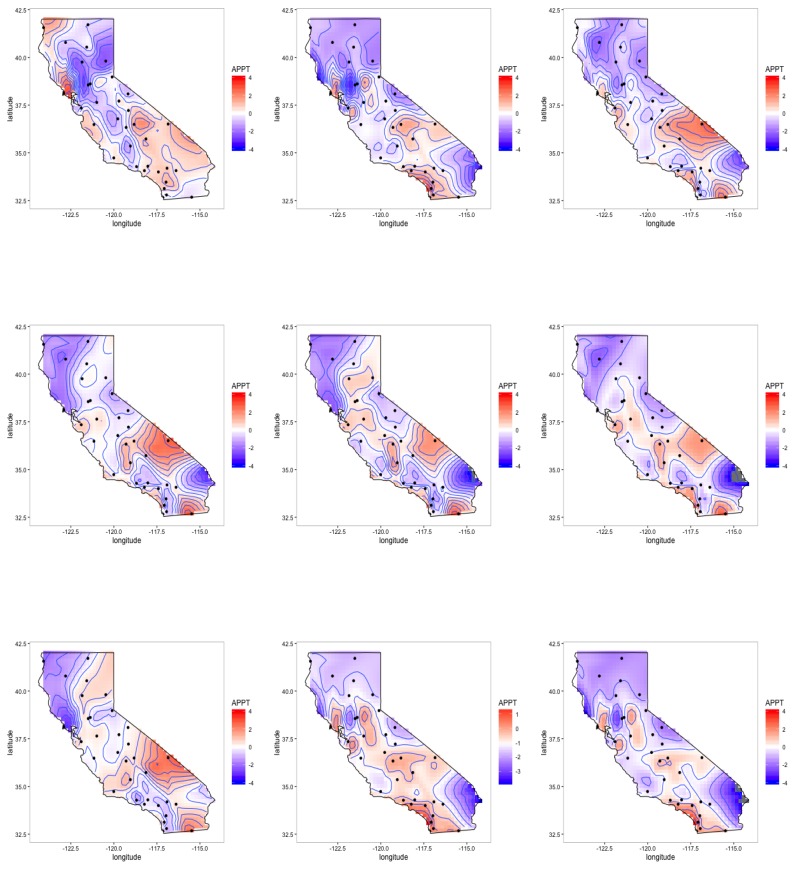
California factor loadings. The $\Lambda_{p}\left(s\right)$ are (**1st row**) Ammonium, Nitrate, Sulfate, (**2nd row**) Total Carbon Mass, Calcium, Iron and (**3rd row**) Potassium, Silicon, and Sulfur. The dots represent the STN and IMPROVE monitoring sites.

The weights of the factors, Λ, are displayed in Figure 5. The factor weights help to inform how much each pollutant contributes to the overall one factor model. The factor loadings in the middle to the southeastern corner and California Bay area are weighed more heavily, suggesting that the one factor model has the greatest concentration of pollutants in those areas. It is important to note that Silicon, Calcium and Sulfate were predominately in the middle section of the state, the main study region where most of the mothers reside. Total carbon mass also appears to contribute to part of the study area, but is more concentrated on west side of the state.

In terms of pollutants and the risk to the fetus, we can compare the one factor model to the pollutants to help infer where the greatest concentrations are in California. The latent factor represents pollutants in the mid to southeastern area, where the concentrations of Ammonium and Sulfate are the highest. This is the area where the majority of the women in the study reside. In the northwestern corner, there is also a pocket of higher concentrations of pollutants near San Francisco where the latent factors represent the concentrations of Potassium, Sulfate and Total Carbon Mass. Although this area is not in the main study area, it is still important, as approximately 15% of the mothers moved residences during the time of the study to the coastal regions, i.e., the San Franscisco area and Los Angeles, which are high pollutant areas. In general, other pollutants such as Iron and Calcium did not contribute much to the latent factor model.

## 5. Conclusions

We constructed a spatiotemporal model that accounts for different sources of speciated pollutants and their association with oral cleft birth defects. Our factor model has advantages over other pollutant models because it accounts for the bias in monitoring stations, STN and IMPROVE, by factoring them into the mean structure of the model. The multivariate spatial dynamic factor model we proposed incorporates factors that are both spatial and temporal through Gaussian processes, unlike some previous work. It provides a framework to assess cleft defects, by weekly averaging speciated pollutants to identify the impacts of pollutants and weeks when the fetus is more susceptible to pollutants, which we note as weeks 3–8. We note that these are not exact time windows as gestational dating in such studies is an estimation and not fixed. We were able to account for mothers’ mobility during pregnancy by factoring in the mothers’ location at the time of gestation and interpolating the pollutants to those locations. We did this by creating an underlying pollutant model that helps to interpolate pollutant levels to mother’s locations and assess the impacts of pollutants on cleft lip and palate defects.

In terms of the health model, we were able to share information across the two types of cleft defects: lip and palate. As cleft lip and cleft palate defects may occur at the same time, we accounted for this correlation in the model. We were able to identify factors that increase the risks of cleft lip and cleft palate defects. We found that mothers with diabetes have an increased risk probability of having a child with a cleft lip or cleft palate defect. The age of the mother also increases the probability of having a cleft defect. In addition, we identified gestation weeks where the fetus is at the greatest risk of birth defects based upon the latent factor of pollutants that mothers were exposed to at the time of gestation. We found that exposure to pollutants during weeks 3 and 6 is associated with an increase in the probability of having a cleft palate defect. With the one factor model, we were able to identify key pollutants in the study region that contributed more to the model than others, in particular Total Carbon Mass and Silicon.

## Figures and Tables

**Table 1:** Cleft defect variables tabulated by maternal and fetal characteristics, cleft lip includes both cleft lip and cleft lip and palate defects; similarly, cleft palate includes both cleft palate and cleft lip and cleft palate defects.

Variable	Levels	% Lip	% Palate	% Control
Age (Mother)	19 and under	26.3	33.3	40.4
	20–24	30.9	29.7	39.4
	25–29	27.8	30.9	41.3
	30+	30.7	36.8	32.5
Education (Mother)	Less Than High School	22.4	22.0	55.6
	High School	21.8	25.9	52.3
	Some College	25.7	22.8	51.5
	College Degree	14.9	17.9	67.1
Fetal Sex	Male	25.1	24.8	50.1
	Female	22.0	21.7	56.3
High Blood Pressure (Mother)	No	20.7	22.2	57.1
	Yes	29.7	26.3	44.0
Smoker	No	21.6	23.7	54.7
	Yes	15.6	18.2	66.2
Alcohol Use	No	21.7	23.7	54.6
	Yes	22.6	25	52.4

**Table 2:** Pearson correlation coefficients of weekly speciated pollutants from stations relevant to the study in the state of California, 2003–2006.

	Nitrate	Sulfate	Total Carbon Mass	Calcium	Iron	Potassium	Silicon	Sulfur
Sulfate	0.41							
Total Carbon Mass	0.38	0.05						
Calcium	0.03	0.08	0.20					
Iron	0.19	0.11	0.48	0.77				
Potassium	0.07	0.17	0.08	0.16	0.12			
Silicon	−0.03	0.01	0.05	0.85	0.78	0.13		
Sulfur	0.41	0.97	0.03	0.10	0.12	0.20	0.04	
Ammonium	0.96	0.60	0.34	0.05	0.19	0.06	−0.01	0.59

**Table 3:** The posterior means, standard deviation (SD), and quantiles for a change in cleft lip defects (top) and cleft palate defects (bottom) in the National Birth Defects Prevention Study, 2003–2007.

	Mean	SD	2.5%	50%	97.5%
Maternal age 20–24 vs. 19 and under	0.540	0.208	0.150	0.545	0.958
Maternal age 25–29 vs. 19 and under	0.556	0.222	0.128	0.562	0.976
Maternal age 30–24 vs. 19 and under	0.605	0.245	0.127	0.609	1.094
Maternal age 35+ vs. 19 and under	0.232	0.285	−0.310	0.217	0.764
Fetus Sex-Female	−0.405	0.134	−0.664	−0.407	−0.157
High school diploma vs. Less than high school	−0.021	0.164	−0.354	−0.019	0.290
Some college vs. Less than high school	0.032	0.173	−0.296	0.027	0.388
College graduate vs. Less than high school	−0.480	0.258	−1.003	−0.469	−0.009
High Blood Pressure	0.332	0.165	0.017	0.330	0.671
Maternal Prediabetes	1.175	0.449	0.261	1.181	2.070
Maternal Smoking	0.089	0.192	−0.310	0.091	0.454
Maternal Alcohol Use	0.049	0.149	−0.243	0.052	0.348
Maternal age 20–24 vs. 19 and under	0.345	0.198	−0.048	0.345	0.760
Maternal age 25–29 vs. 19 and under	0.439	0.208	0.027	0.437	0.866
Maternal age 30–34 vs. 19 and under	0.440	0.231	−0.018	0.428	0.899
Maternal age 35+ vs. 19 and under	0.385	0.268	−0.139	0.381	0.895
Fetus Sex-Female	−0.247	0.133	−0.503	−0.246	0.021
High School Education vs. Less than high school	0.023	0.150	−0.262	0.026	0.309
Some college vs. Less than high school	−0.109	0.169	−0.426	−0.114	0.209
College graduate vs. Less than high school	−0.430	0.167	−0.943	−0.412	−0.001
High Blood Pressure	0.05	0.155	−0.221	0.007	0.332
Maternal Prediabetes	1.214	0.431	0.391	1.196	2.072
Maternal Smoking	0.183	0.174	−0.153	0.190	0.510
Maternal Alcohol Use	0.106	0.144	−0.191	0.109	0.390

## Appendix A. Factor Model Parameter Priors and Updates

Parameters are updated using a Markov Chain Monte Carlo (MCMC) scheme. Denote the full conditional distribution of a parameter θ as [θ].

Observation Means ($\mu_{p}$): For each pollutant p, assume independent prior distributions, $\mu_{p}∼N\left(m_{0},s_{0}^{2}\right)$.

Let $Y^{p}={\left(Y_{1}^{p},Y_{2}^{p},\cdots ,Y_{T}^{p}\right)}^{\prime }$ be a NT×1 vector of pollutant p for all locations and time points, $\Lambda^{p}=\left(\Lambda_{1}^{p},\cdots ,\Lambda_{m}^{p}\right)$ be a N×mN matrix of factor loadings, $\Lambda^{p}$ be a NT×mNT block diagonal matrix with each of the T diagonal blocks equal to $\Lambda^{p}$, and $\delta ={\left(\delta_{1},\cdots ,\delta_{T}\right)}^{\prime }$ be a mNT×1 vector of all factors across all time. Then, we can rewrite the conditional distribution as $Y^{p}|\mu_{p},\delta ,\Lambda^{p},\sigma_{p}^{2}∼N\left(\mu^{p}1_{\mathrm{NT}}+\Lambda^{p}\delta ,\sigma_{p}^{2}I_{\mathrm{NT}}\right)$.

The resulting full conditional will then be, $\left[\mu_{p}\right]∼N\left(\left(m_{0}/s_{0}^{2}+1_{\mathrm{NT}}^{\prime }\left(Y^{p}-\Lambda^{p}\delta \right)/\sigma_{p}^{2}\right){\left(1/s_{0}^{2}+\mathrm{NT}/\sigma_{p}^{2}\right)}^{-1},{\left(1/s_{0}^{2}+\mathrm{NT}/\sigma_{p}^{2}\right)}^{-1}\right)$.

Observation Variances ($\sigma_{p}^{2}$): For each pollutant p, assume independent prior distributions, $\sigma_{p}^{2}∼\mathrm{IG}\left(n_{\sigma }/2,n_{\sigma }s_{\sigma }/2\right)$.

Referring to the notation defined above, the resulting full conditional will be $\left[\sigma_{p}^{2}\right]∼\mathrm{IG}\left(\left(\mathrm{NT}+n_{\sigma }\right)/2,\left({\left(Y^{p}-\mu^{p}1_{\mathrm{NT}}-\Lambda^{p}\delta \right)}^{\prime }\left(Y^{p}-\mu^{p}1_{\mathrm{NT}}-\Lambda^{p}\delta \right)+n_{\sigma }s_{\sigma }\right)/2\right).$

Factor Variances ($\tau_{f}^{2}$): For each factor f, assume independent prior distributions, $\tau_{f}^{2}∼\mathrm{IG}\left(n_{\tau }/2,n_{\tau }s_{\tau }/2\right)$.

Let $\Gamma_{f}$ be a NT×NT block diagonal matrix with each of the T diagonal blocks equal to $\Gamma_{f}$, $\delta_{f}={\left(\delta_{f,1},\cdots ,\delta_{f,T}\right)}^{\prime }$ be a NT×1 vector of factor f for t=1,⋯,T, and $\delta_{f}^{*}={\left(\delta_{f,0},\cdots ,\delta_{f,T-1}\right)}^{\prime }$ be a NT×1 vector of factor f for t=0,⋯,T−1.

The resulting full conditional will be, $\left[\tau_{f}^{2}\right]∼\mathrm{IG}\left(\left(\mathrm{NT}+n_{\tau }\right)/2,\left({\left(\delta_{f}-\Gamma_{f}\delta_{f}^{*}\right)}^{\prime }\left(\delta_{f}-\Gamma_{f}\delta_{f}^{*}\right)+n_{\tau }s_{\tau }\right)/2\right)$.

Spatially Varying Factor Loadings ($\lambda_{f}^{p,s}$): For each factor-pollutant pair, assume a Gaussian process prior for the factor loadings, diag($\Lambda_{f}^{p})∼N\left(0,s_{\Lambda_{f,p}}^{2}R\left(\phi_{\Lambda_{f,p}}\right)\right)$. Let $\Sigma_{p}$ be the block diagonal matrix of the m covariance matrices associated with the m factor loadings for pollutant p.

Let $\delta_{\mathrm{diag},t}=\left(\mathrm{diag}\left(\delta_{1,t}\right),\cdots ,\mathrm{diag}\left(\delta_{m,t}\right)\right)$ a N×mN matrix of the spatial factors at time t, $\delta_{\mathrm{diag}}$ a NT×mN matrix of the $\delta_{\mathrm{diag},t}$ stacked on top of each other for t=1,⋯,T, and $\Lambda_{\mathrm{vec}}^{p}={\left(\lambda_{1}^{p}\left(s_{1}\right),\cdots ,\lambda_{1}^{p}\left(s_{N}\right),\cdots ,\lambda_{m}^{p}\left(s_{1}\right),\cdots ,\lambda_{m}^{p}\left(s_{N}\right)\right)}^{\prime }$ a mN×1 vector of the factor loadings at all locations for pollutant p. Then, we can rewrite the conditional distribution as $Y^{p}|\mu_{p},\delta ,\Lambda^{p},\sigma_{p}^{2}∼N\left(\mu_{p}1_{\mathrm{NT}}+\delta_{\mathrm{diag}}\Lambda_{\mathrm{vec}}^{p},\sigma_{p}^{2}I_{\mathrm{NT}}\right).$

Using standard multivariate Normal theory, the resulting full conditional will be, $\left[\Lambda_{\mathrm{vec}}^{p}\right]∼N\left(\mu_{\Lambda^{p}},\Sigma_{\Lambda^{p}}\right)$ where $\Sigma_{\Lambda^{p}}={\left(\Sigma_{p}^{-1}+1/\sigma_{p}^{2}\delta_{\mathrm{diag}}^{\prime }\delta_{\mathrm{diag}}\right)}^{-1}$ and $\mu_{\Lambda^{p}}=1/\sigma_{p}^{2}\Sigma_{\Lambda^{p}}\left(\delta_{\mathrm{diag}}^{\prime }\left(Y^{p}-\mu_{p}1_{\mathrm{NT}}\right)\right)$.

Spatially Constant Factor Loadings ($\lambda_{f}^{p}$): For each pollutant p assume independent prior distributions, $\lambda^{p}={\left(\lambda_{1}^{p},\cdots ,\lambda_{m}^{p}\right)}^{\prime }∼N\left(0,\sigma_{\lambda^{p}}^{2}I_{m}\right).$

Let $\delta_{\mathrm{mat}}=\left(\delta_{1},\cdots ,\delta_{m}\right)$ be the NT×m matrix of factors across time.

Then, the resulting full conditional will be, $\left[\lambda^{p}\right]∼N\left(\mu_{\lambda^{p}},\Sigma_{\lambda^{p}}\right)$ where $\Sigma_{\lambda^{p}}={\left(1/\sigma_{\lambda^{p}}^{2}I_{m}+1/\sigma_{p}^{2}\delta_{\mathrm{mat}}^{\prime }\delta_{\mathrm{mat}}\right)}^{-1}$ and $\mu_{\lambda^{p}}=\Sigma_{\lambda^{p}}\left(1/\sigma_{p}^{2}\delta_{\mathrm{mat}}^{\prime }\left(Y^{p}-\mu_{p}1_{\mathrm{NT}}\right)\right)$.

Factor Evolution Coefficients ($\gamma_{f}^{s}$): To ensure stationarity in time, the factor evolution coefficients are restricted to the interval (−1,1), so assume a Truncated Gaussian process prior, $\gamma_{f}∼{\mathrm{TN}}_{\left(-1,1\right)}\left(0_{N},s_{\Gamma_{f}}^{2}R\left(\phi_{\Gamma_{f}}\right)=\Sigma_{\Gamma_{f}}\right)$.

Let $\gamma_{f}=\mathrm{diag}\left(\Gamma_{f}\right)$ be a N×1 vector of evolution coefficients, $\delta_{f,\mathrm{diag}}^{*}$ be the NT×N matrix stacking T diagonal N×N matrices of $\delta_{f,t}$ for t=0,⋯,T−1.

Then, the resulting full conditional will be $\left[\gamma_{f}\right]∼{\mathrm{TN}}_{\left(-1,1\right)}\left(\mu_{\gamma_{f}},\Sigma_{\gamma_{f}}\right)$, where $\Sigma_{\gamma_{f}}={\left(\Sigma_{\Gamma_{f}}^{-1}+1/\tau_{f}^{2}\delta_{f,\mathrm{diag}}^{{*}^{\prime }}\delta_{f,\mathrm{diag}}^{*}\right)}^{-1}$ and $\mu_{\gamma_{f}}=\Sigma_{\gamma_{f}}\left(1/\tau_{f}^{2}\delta_{f,\mathrm{diag}}^{{*}^{\prime }}\delta_{f}\right).$

Factors ($\delta_{t}$): Assume $\delta_{f,0}∼N\left(0_{N},s_{\delta_{f}}^{2}R\left(\phi_{\delta_{f}}\right)\right)$ is a realization from a Gaussian process for each factor f. Let $m_{0}=0_{\mathrm{mN}}$ and $C_{0}$ be an mN×mN block diagonal matrix corresponding to the covariance matrix for the collection of all factors at time 0, $\delta_{0}={\left(\delta_{1,0},\cdots ,\delta_{m,0}\right)}^{\prime }$. Let $\Sigma_{\epsilon }$ and $\Sigma_{w}$ be the diagonal covariance matrices associated with $\epsilon_{t}$ and $w_{t}$, respectively.

The factors are updated through a Forward Filtering Backwards Sampling (FFBS) algorithm [21,22]:

**Forward Filtering:** For t=1,⋯,T, compute $m_{t}=a_{t}+A_{t}\left(Y_{t}-{\tilde{Y}}_{t}\right)$ and $C_{t}=R_{t}-A_{t}Q_{t}A_{t}^{\prime }$, where $a_{t}=\Gamma m_{t-1}$, $A_{t}=R_{t}\Lambda^{\prime }Q_{t}^{-1}$, $Q_{t}=\Lambda R_{t}\Lambda^{\prime }+\Sigma_{\epsilon }$, $R_{t}=\Gamma C_{t-1}\Gamma^{\prime }+\Sigma_{w}$, and ${\tilde{Y}}_{t}=\mu +\Lambda a_{t}$. Then, sample $\delta_{T}∼N\left(m_{T},C_{T}\right)$.
**Backwards Sampling:** For t=(T−1),⋯,0 sample $\delta_{t}∼N\left({\tilde{a}}_{t},{\tilde{C}}_{t}\right),$ where ${\tilde{a}}_{t}=m_{t}+B_{t}\left(\delta_{t+1}-a_{t+1}\right)$, ${\tilde{C}}_{t}=C_{t}-B_{t}R_{t+1}B_{t}^{\prime }$, and $B_{t}=C_{t}\Gamma^{\prime }R_{t+1}^{-1}$.

When data are unobserved at a particular time, t, we assume it is missing at random. Following the reasoning of [23], there is no additional information incorporated into the posterior and the factors are updated setting $m_{t}=a_{t}$ in the FFBS algorithm.

Spatial Parameters ($s_{i}^{2},\phi_{i}$): There are mP parameters $s_{\Lambda_{f,p}}^{2}$, m parameters $s_{\Gamma_{f}}^{2}$ and m parameters $s_{\delta_{f}}^{2}$ describing the variance in the spatial processes. Denote these as $s_{i}^{2}$ with priors $\mathrm{IG}(n_{i}/2,n_{i}s_{i}/2)$. Similar for the range parameters $\phi_{i}$ with priors $\mathrm{IG}(a_{i},b_{i})$.
